# Significant Improvement of Thermal Stability for CeZrPrNd Oxides Simply by Supercritical CO_2_ Drying

**DOI:** 10.1371/journal.pone.0088236

**Published:** 2014-02-07

**Authors:** Yunzhao Fan, Zizi Wang, Ying Xin, Qian Li, Zhaoliang Zhang, Yingxia Wang

**Affiliations:** 1 School of Chemistry and Chemical Engineering, University of Jinan, Jinan, P. R. China; 2 State Key Laboratory for Rare Earth Materials Chemistry and Applications, Peking University, Beijing, P. R. China; Queen’s University Belfast, United Kingdom

## Abstract

Pr and Nd co-doped Ce-Zr oxide solid solutions (CZPN) were prepared using co-precipitation and microemulsion methods. It is found that only using supercritical CO_2_ drying can result in a significant improvement of specific surface area and oxygen storage capacity at lower temperatures for CZPN after aging at 1000°C for 12 h in comparison with those using conventional air drying and even supercritical ethanol drying. Furthermore, the cubic structure was obtained in spite of the fact that the atomic ratio of Ce/(Ce+Zr+Pr+Nd) is as low as 29%. The high thermal stability can be attributed to the loosely aggregated morphology and the resultant Ce enrichment on the nanoparticle surface, which are caused by supercritical CO_2_ drying due to the elimination of surface tension effects on the gas-liquid interface.

## Introduction

Cerium dioxide (CeO_2_) is an indispensable oxygen storage capacity (OSC) material in three-way catalysts (TWCs), which work close to stoichiometric conditions so as to eliminate simultaneously CO, NO_x_ and hydrocarbons contained in automotive exhausts [1]. This is due to the ability of CeO_2_ to store and release oxygen under lean and rich conditions respectively, according to the unique redox behavior between Ce^4+^ and Ce^3+^. However, the increasingly stringent standards for the automotive emissions have set new challenges for the development of more thermally stable oxygen storage materials which can withstand temperatures at or above 1000°C for long periods of time [2]. The current TWCs are based on Ce-Zr oxide solid solutions (CZ) [3]. Generally, two strategies were adopted to improve the thermal stability of CZ. One approach is doping with other metal ions into the ceria lattice. Therein, Al [4] and Pr/Nd [5] are mostly used. The composite oxides of Al_2_O_3_ and CZ in intervening layers on a nanometer scale show higher surface area and OSC after heat treatment at 1000°C due to the role of the so-called “diffusion barrier” of Al_2_O_3_; however, the presence of bulk alumina and/or CeAlO_3_, which are deleterious to the OSC, was generally concomitant [2]. Comparatively, the doping of Pr or Nd causes the lattice deformation of the tetragonal Zr-rich mixed oxides to form a pseudocubic structure and prevents the phase segregation after calcinations at 1050°C for 5 h [5]. In view of these facts, it is preferred that the CZ is first doped with Pr/Nd and then suspended with Al_2_O_3_, and finally washcoated on the honeycomb.

The other approach to improve thermal stability of CZ is changing preparation methods, which include co-precipitation, sol–gel, solution combustion, surfactant-assisted and microemulsion [6]. However, the drying method used to remove solvents of the wet precipitates, which would affect many important powder properties, for example, the homogeneity, the size of the agglomerates/aggregates and the extent of agglomeration/aggregation, is scarcely studied. Often this process is carried out conventionally in air/vacuum, which results in particle agglomerates by the capillary attraction because of the existence of the liquid to vapor phase transformation of the solvent. To solve this problem, supercritical ethanol drying technology was reported for the preparation of La-doped CZ, which shows better thermal stability (higher surface areas and larger OSCs) in comparison with the conventional drying techniques [7], [8]. The authors attributed this to the elimination of vapor–liquid interface in the process of supercritical drying. However, the following questions still exist: (1) drastic conditions are required (critical point of ethanol: 243°C, 6.3 MPa). The same case applies to using supercritical water [9], [10]; (2) the structure is tetragonal (The molar ratio of Ce/Zr equals to 1/4). It is well known that preserving the cubic c phase is beneficial for the OSCs and catalytic activity, while the appearance of the tetragonal t phase is detrimental [11], [12]. Looking for milder reaction conditions, supercritical CO_2_ can be used as an alternative due to its easily accessible critical point (31°C, 7.4 MPa) and nontoxic, nonflammable, inexpensive and recyclable nature. Indeed, a lot of materials including CeO_2_ [13], CeO_2_–Al_2_O_3_ [14] and ZrO_2_ [15]–[17] have been prepared using the supercritical CO_2_ process. Unfortunately, the only paper concerning CZ prepared by supercritical CO_2_ drying reported the experimental temperature and pressure are 455°C and 19.5 MPa, respectively [18]. It is unimaginable that the temperature is so much higher than the critical point of CO_2_.

**Figure 1:**
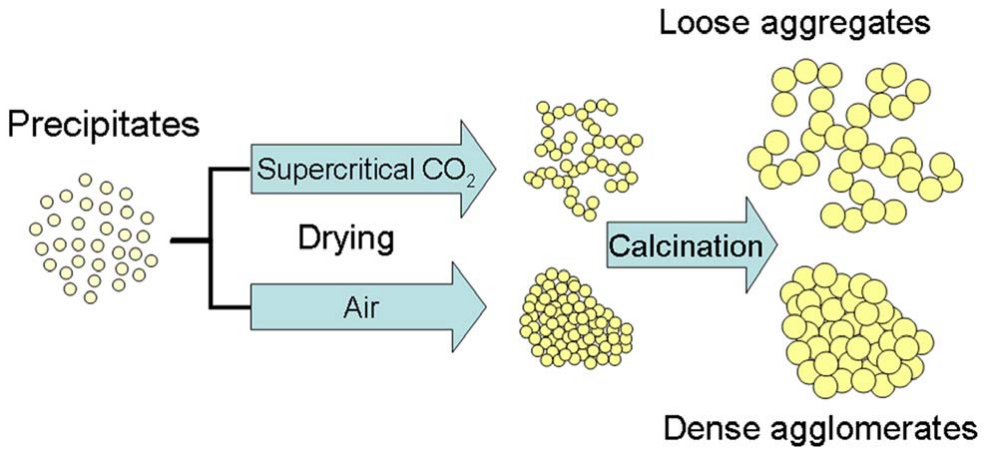
The schematic representation of the synthesis steps to produce thermally stable CZPN.

The improvement of thermal stability is reflected in high surface areas and high porosity at high temperatures, which are desired for practical applications taking into consideration the potential kinetic advantages. In this paper, Pr and Nd co-doped CZ (CZPN) was prepared using co-precipitation (CO) and microemulsion (ME) methods followed by drying at 100°C in air, and co-precipitation followed by supercritical CO_2_ drying (CO–SC) and microemulsion followed by supercritical CO_2_ drying (ME–SC) (50°C, 15 MPa). Surprisingly, after aging at 1000°C for 12 h, the significant improvement of surface areas and OSCs at lower temperatures were obtained for both SC samples in comparison with those using supercritical ethanol and conventional drying. Furthermore, the cubic structure was obtained in spite of the fact that the atomic ratio of Ce/(Ce+Zr+Pr+Nd) is as low as 29% in CZPN (The actual contents of Ce, Zr, Pr and Nd were determined by X-ray fluorescence spectrometry (XRF) and are given in Table 1, nearly in accordance with the nominal values, suggesting that the drying method cannot change the compositions). The schematic representation of the general synthesis steps to produce thermally stable CZPN is shown in Figure 1.

**Table 1:** Textural properties and compositions.

Samples	BET surfacearea (m^2^/g)	Pore volume(cm^3^/g)	Average porediameter (nm)	Unit cellparameter (Å)	Unit cellvolume (Å^3^)	Crystallitesize (nm)	Ce/Zr/Pr/Nd(molar)
CO	9.71 (9.71)^a^	0.025 (naught)^b^	9.5	5.273(3)^c^	146.7(2)^c^	9.53	26.3/59.9/7.3/6.5
CO–SC	52.4 (47.7)^a^	0.379 (0.0022)^b^	25.5	5.274(2)^c^	146.7(2)^c^	8.43	33.8/48.1/9.1/9.0
ME	29.4 (27.4)^a^	0.135 (0.0008)^b^	15.3	5.272(1)^c^	146.56(7)^c^	12.17	27.9/58.0/7.4/6.7
ME–SC	63.3 (56.3)^a^	0.479 (0.0032)^b^	26.5	5.272(1)^c^	146.51(8)^c^	8.57	33.4/51.6/7.9/7.1

a t-plot external surface area;

b t-plot micropore volume;

c Calculated standard deviations from Le Bail fitting are given in parentheses.

## Materials and Methods

### Synthesis

CZPN mixed oxides were prepared by co-precipitation (CO), microemulsion (ME), co-precipitation followed by supercritical CO_2_ (CO–SC) and microemulsion followed by supercritical CO_2_ (ME–SC). In the as-prepared samples, the atom ratios of Ce, Pr and Nd to Zr are 0.51, 0.12 and 0.12, respectively. In the CO synthesis, NH_3_·H_2_O (25%) and H_2_O_2_ were dropped into a stoichiometric solution (0.2 mol/L) of Ce(NO_3_)_3_·6H_2_O, Zr(NO_3_)_4_·3H_2_O, Pr(NO_3_)_3_·6H_2_O and Nd(NO_3_)_3_·6H_2_O under vigorous agitation and then the resultant precipitate was aged in air for 24 h at room temperature and pressure. After filtration, the aerogels were dried at 100°C overnight and calcined at 1000°C for 12 h in air. In the ME synthesis [19], the microemulsion (I) of Ce(NO_3_)_3_·6H_2_O, Zr(NO_3_)_4_·3H_2_O, Pr(NO_3_)_3_·6H_2_O and Nd(NO_3_)_3_·6H_2_O, polyethylene glycol octylphenyl ether (as surfactant), cyclohexane (as oil phase) and 1-hexanol (as cosurfactant) was prepared; the microemulsion (II) of ammonia, polyethylene glycol octylphenyl ether, cyclohexane, 1-hexanol and hydrogen peroxide (as oxidant) was prepared; the reverse microemulsion (I) was mixed with (II) to react. The aerogels were filtered, washed and dried overnight at 100°C and calcined at 1000°C for 12 h in air. In the SC processes, water in the aerogels was first exchanged with anhydrous ethanol. Then the cake was loaded in a stainless-steel reactor to supercritical CO_2_ drying (15 MPa at 50°C in order to insure the homogenous phase of CO_2_ and ethanol and thus eliminate the gas-liquid interface [20]) and maintained under flow for 5 h. After that, the pressure was slowly released. Finally, the aerogels were calcined at 1000°C for 12 h in air.

### Characterization

The elemental compositions were measured by XRF (ARL–9800, Switzerland). X-ray diffraction (XRD) patterns were recorded on a Rigaku D/max–2500/PC diffractometer employing Cu Kα radiation (λ = 1.5418 Å) operating at 50 kV and 200 mA. A Le Bail method was used for the profile fitting of the XRD patterns. Raman spectra were measured using a Raman spectroscope (HR800) with a CCD camera. The 632.8 nm line of a He–Ne laser was used to simulate the Raman spectra. The measurements were carried out with a microscope by using a ×50 objective lens (focus diameter larger than 1 micron) and the data are recorded in a backscattering geometry. The Brunauer–Emmett–Teller (BET) surface area and pore structure were measured by N_2_ adsorption/desorption using a Micromeritics 2020M instrument. Before N_2_ physisorption, the sample was outgassed at 300°C for 5 h. Field-emission scanning electron microscopy (FESEM) was conducted on a JEOL SU70. Transmission electron microscopy (TEM) equipped with selective area electron diffraction (SAED) was conducted on a JEOL JEM–2010 microscope at an accelerating voltage of 200 kV. X-ray photoelectron spectroscopy (XPS) data were obtained on an AXIS-Ultra instrument from Kratos Analytical using monochromatic Al Kα radiation (225 W, 15 mA, 15 kV) and low-energy electron flooding for charge compensation. To compensate for surface charge effects, the binding energies were calibrated using the C 1 s hydrocarbon peak at 284.80 eV.

### Performance Studies

Temperature-programmed reduction with H_2_ (H_2_–TPR) experiments were performed in a quartz reactor with a thermal conductivity detector (TCD) to monitor the H_2_ consumed. A 50 mg sample was pretreated in situ at 500°C for 1 h in a flow of O_2_ and cooled to room temperature in the presence of O_2_. TPR was conducted at 10°C/min up to 900°C in a 30 mL/min flow of 5 vol.% H_2_ in N_2_. After the first cycle, the sample was cooled to room temperature in the H_2_/N_2_ mixture. The sample was then reoxidized at 500°C for 1 h in O_2_ and cooled to room temperature in O_2_, and then a second TPR run was conducted. Similar consecutive TPR runs were carried out three times. To quantify the total amount of H_2_ consumed, CuO was used as a calibration reference. The total OSC was measured at low temperatures (200°C) and high temperatures (700°C). In respect of the former, the 50 mg sample was first reduced at 550°C in 5 vol.% H_2_ in N_2_ flow for 40 min. After cooling down to 200°C in He flow, the high-purity O_2_ pluses were injected up to the breakthrough point and the O_2_ consumption was measured. As to the latter, the H_2_ consumption was measured. The 50 mg sample was first oxidized at 500°C in O_2_ flow for 30 min. After heating up to 700°C in He flow, the high-purity H_2_ pluses were injected up to the breakthrough point.

## Results and Discussion

**Figure 2:**
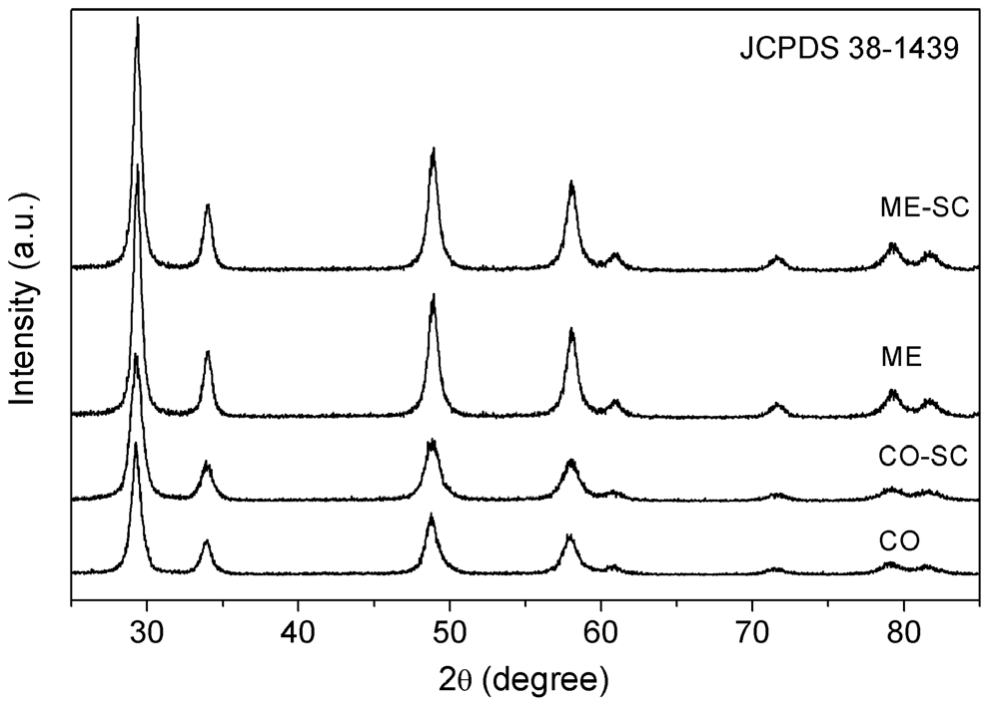
XRD patterns of CO, CO–SC, ME and ME–SC.

Although the atomic ratio of Ce/(Ce+Zr+Pr+Nd) equals 29%, the XRD patterns of all CZPN samples (Figure 2) show a cubic fluorite structure and no peak splitting that would indicate the presence of two phases could be detected. The peaks are symmetrical, suggesting sample homogeneity. Single-phase and homogeneous materials are preferred to phase-segregated ones (for example CZ–0.75 in Figure S1) since it is believed that this results in better thermal stability and redox properties [2]. The unit cell parameters (*a*) derived from Le Bail fitting of XRD (Table 1, Figure S2 and Table S1) is nearly the same, in agreement with the formation of a solid solution. However, the crystallite sizes calculated according to Scherrer equation for SC samples are lower than those using conventional drying. This suggests that supercritical CO_2_ prevents particle agglomeration and coarsening. A smaller particle size increases the solubility of zirconia in cubic ceria, which is beneficial to the OSC [21]. Moreover, the grain size influences the defect formation energy, and in particular the concentration of oxygen vacancies. That is, the smaller the grain size, the more the oxygen vacancy and the oxygen release ability [5].

**Figure 3:**
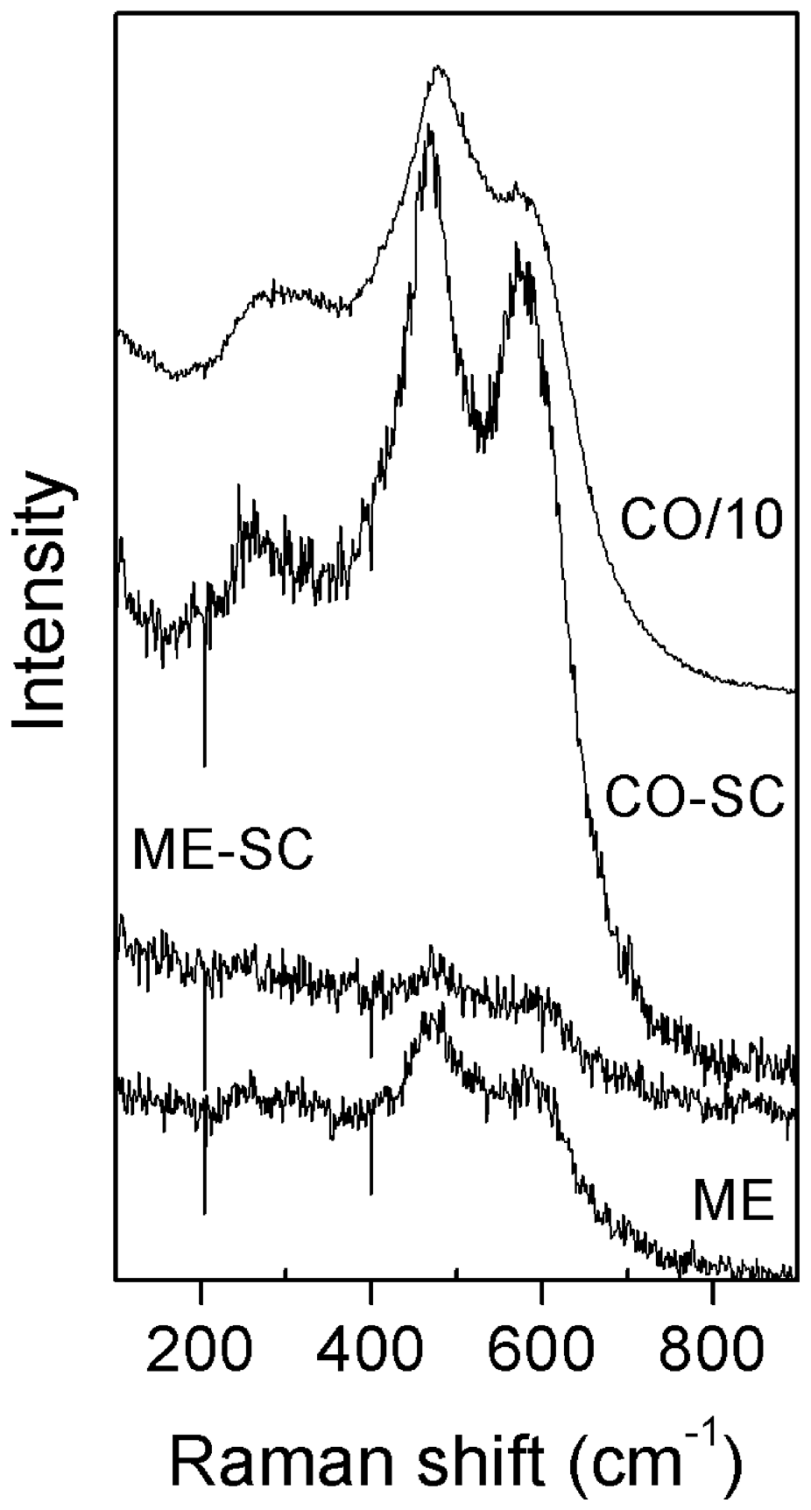
Raman spectra of CO, CO–SC, ME and ME–SC.

The phase composition was further investigated by Raman spectroscopy (Figure 3). The band observed at ∼463 cm^−1^ can be assigned to the symmetric breathing mode of the O atoms around each Ce^4+^ because that is the only allowed Raman mode with F_2 g_ symmetry in metal oxides with a fluorite structure [22]. The peaks become broader for ME and ME–SC, which can be explained by serious lattice distortions due to the incorporation of Pr, Nd and Zr into cubic CeO_2_ (*c* phase) [22]. However, the coexistence of the 463 cm^−1^ peak coupled with a peak at ∼300 cm^−1^ suggests the presence of pseudocubic phase (*c*′ which contains *c* and *t*″) for CO and CO–SC [21]. The *t*″ phase is a tetragonal metastable phase; however, it does not show any tetragonality (the axial ratio equals to 1) and thus is indistinguishable by XRD from the *c* phase [11]. Based on Raman spectra, it can be concluded that a more homogeneous structure was obtained in ME and ME–SC in comparison with CO and CO–SC. In addition, the peak at ∼594 cm^−1^ is characteristic of oxygen vacancies in the cubic lattice [22], which is generated by incorporation of hetero atoms.

For TWC application, besides the homogeneity of the mixed oxide, textural stability is fairly important. Pr and Nd co-doping did improve the BET surface area (Table 1 and Table S2). Furthermore, the BET surface areas and pore volumes for SC samples are much larger than those using conventional drying (Table 1) and supercritical ethanol drying (Figure S3 and Table S2). In the case of CO samples, the surface area of CO–SC is 5.4 times as large as that of CO, while the pore volume is 15.2 times as large as that of CO. As for ME samples, the surface area and pore volume for ME–SC are 2.2 and 3.5 times as large as those of ME, respectively.

**Figure 4:**
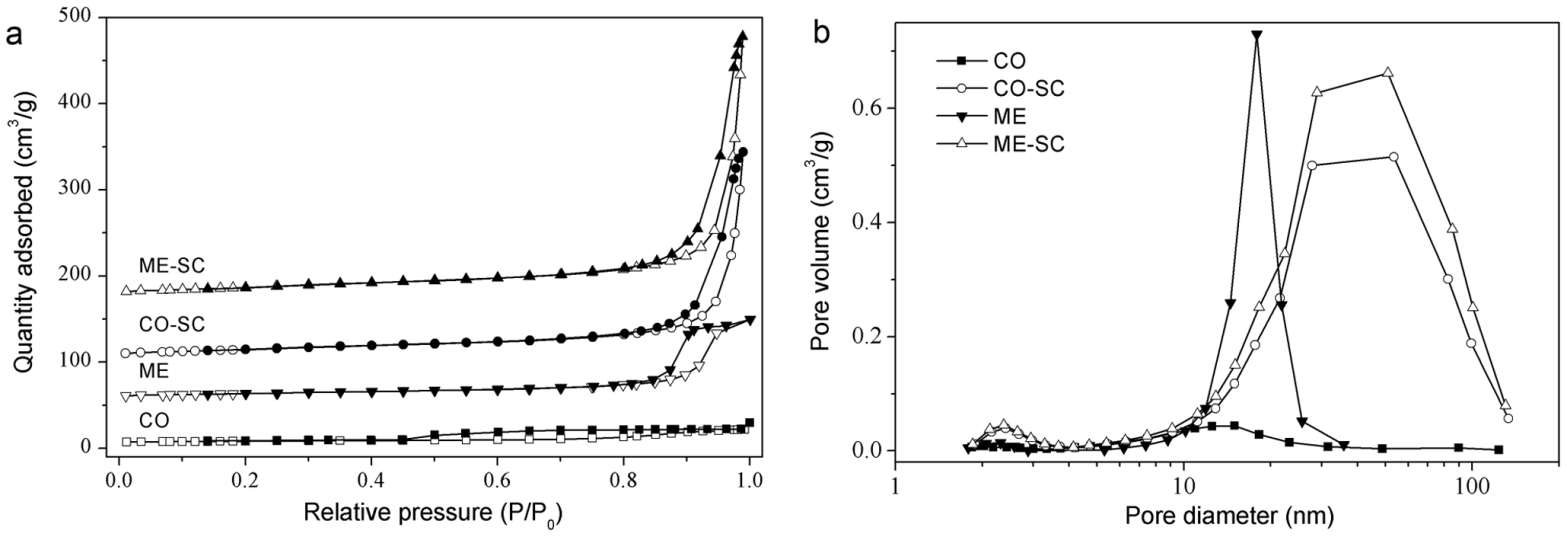
N_2_ adsorption/desorption isotherms (a) and pore size distribution plots (b) for CO, CO–SC, ME and ME–SC.

The negligible pore for CO (Table 1 and Figure 4) suggests the complete sintering by high-temperature aging. The adsorption-desorption isotherms of ME (Figure 4a) show type IV isotherm (IUPAC classification) with type H1 hysteresis loop, which is associated with pores known to consist of agglomerates, and hence narrow pore distributions and smaller pore sizes were formed (Figure 4b). The isotherms of CO–SC and ME–SC (Figure 4a) comply with type II with a type H3 hysteresis loop in the relative pressure (p/p_0_) range of 0.8–1.0, which is observed with aggregates of plate-like particles giving rise to slit-shaped pores [23]. This corresponds to a shift in the pore size distribution to higher pore diameters and the presence of macropores (>50 nm). As shown in Figure 4b, there were two pore size distributions: one small peak centered at 2.4 nm and one strong and broad peak centered at about 50 nm. In addition, the *t*-plot micropore volumes for SC samples are much larger than those of CO and ME though the values are extremely low. All these facts confirm the more loose texture for the SC samples due to the absence of surface tension in supercritical CO_2_, which is the reason for the wide pore size distribution and the increase in surface areas [24].

**Figure 5:**
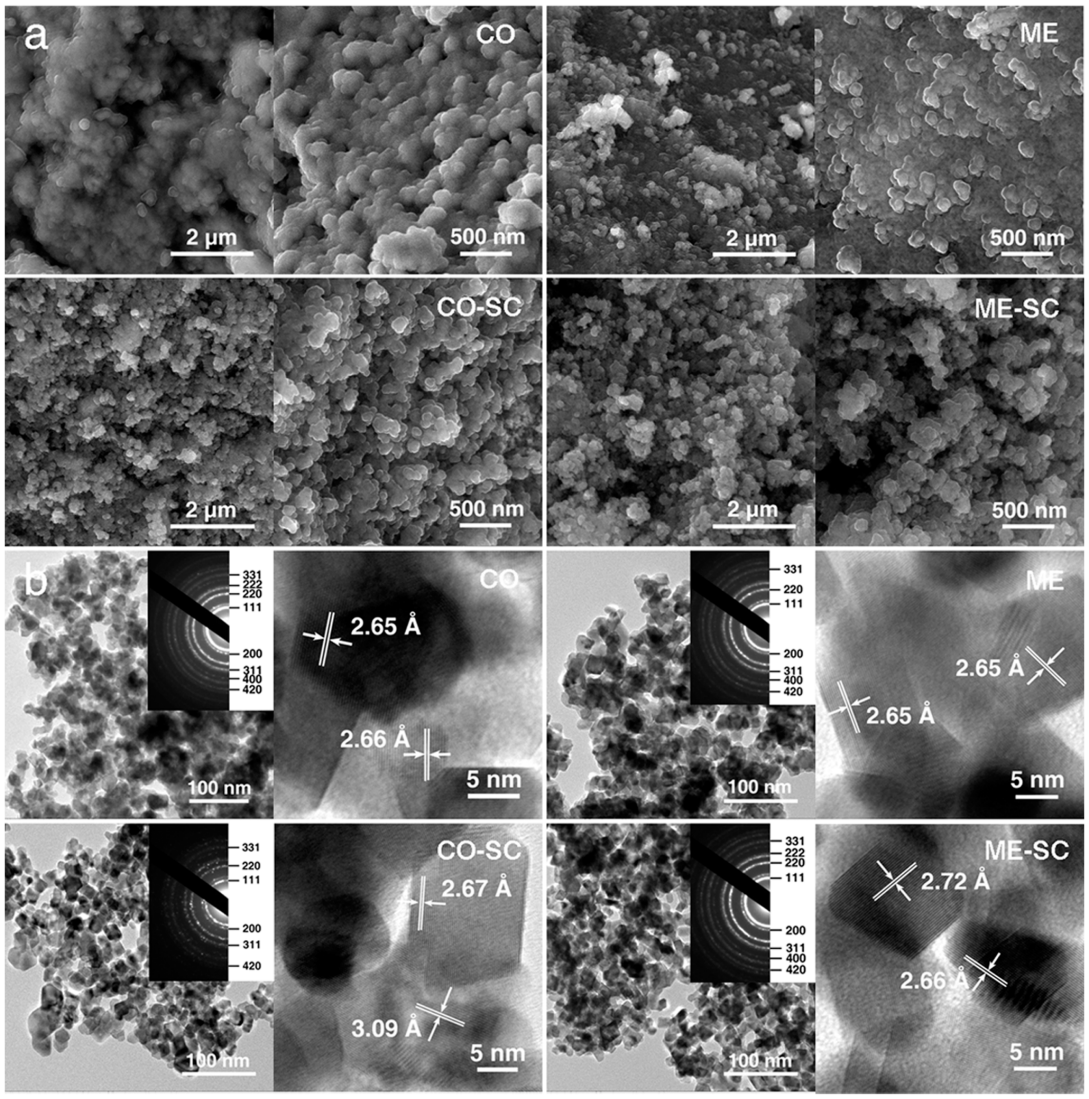
SEM images (a); TEM and HRTEM images (b) as well as the corresponding SAED patterns (insets) for CO, CO–SC, ME and ME–SC.

The morphology is first checked by FESEM (Figure 5a). The sintering and aggregation of primary particles were severe for CO and thus negligible pores were formed. In the case of ME, the situation is improved partially. The interstitial space among nanoparticles in the agglomeration constitutes mesopores. As expected in Figure 1, the loosely aggregated morphology was obtained for both CO–SC and ME–SC, which is attributed to the weak interaction between the oxide nanoparticles during the supercritical drying process [9], [10]. The morphology observation is direct evidence of the loose aggregate structures for CO–SC and ME–SC.

The primary particles of CZPN around 10 nm can be distinguished by TEM images (Figure 5b), consistent with the XRD results (Table 1). The corresponding SAED patterns and high resolution TEM (HRTEM) reinforced the cubic structure.

It is reported that the higher the surface area, the larger the number of surface sites the sample can provide, and thus the better the catalytic activities the sample will have. In this paper, H_2_–TPR and total OSCs were used to evaluate the performance.

**Figure 6:**
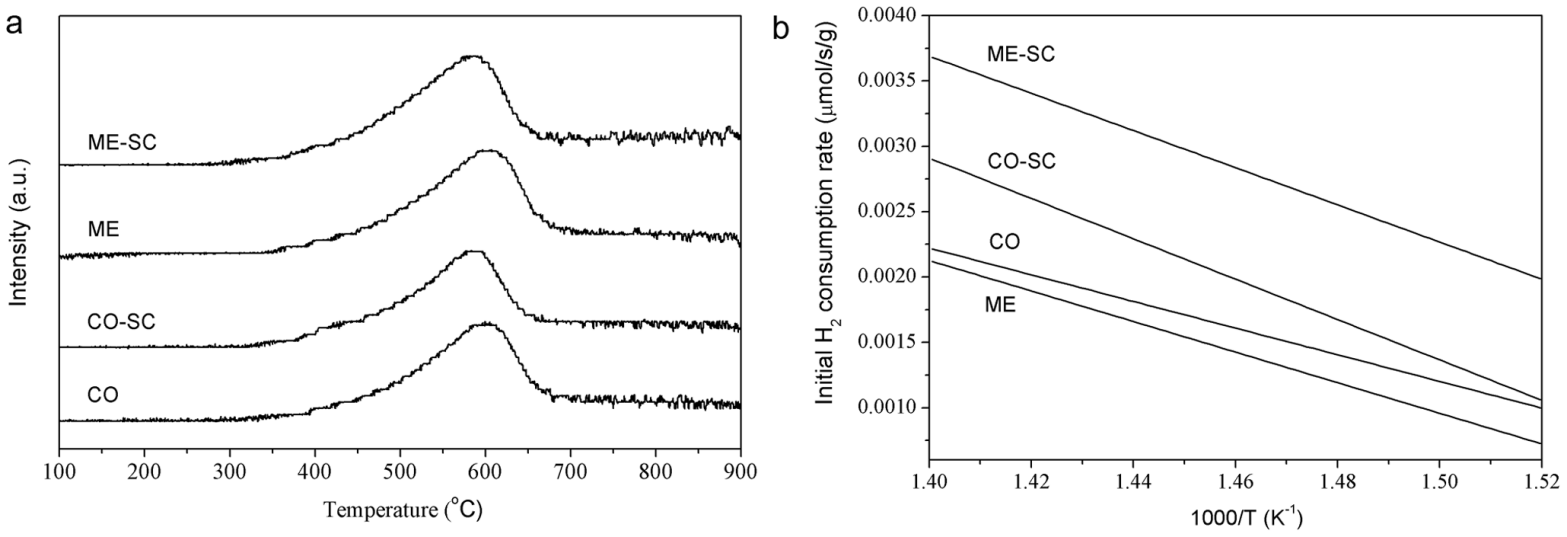
H_2_–TPR spectra (a) and initial H_2_ consumption rate per gram sample (b) for CO, CO–SC, ME and ME–SC during the first cycle.

As shown in Figure 6a, only one broad peak was observed for all CZPN samples. In contrast, CeO_2_ shows two peaks centered at about 500°C and 800°C, which can be assigned to the reduction of surface oxygen and lattice oxygen, respectively [25]. Furthermore, both CO–SC and ME–SC show lower peak temperatures and higher H_2_ consumption than CO and ME, respectively (Table 2). This is clearly demonstrated by the initial H_2_ consumption rate per gram of samples where reduction is less than 5% (Figure 6b) [26]. These facts suggest that the CZPN samples prepared by SC possess a higher redox property than those prepared by the conventional drying method. Furthermore, as shown in Figure S4 and Table S3, the cyclic TPR profile characteristics were almost reproducible, indicating that the oxidative-reductive reaction is reversible. The improved reducibility was displayed by the total OSC data (Table 2). The OSCs for CO–SC and ME–SC are higher than that for CO and ME, respectively. This improvement is remarkable especially at lower temperatures (200°C). From Table 2, it is also observed that the OSCs at 200°C and BET surface areas have the same tendency, suggesting that the redox activity is dependent on surface areas at lower temperatures, because the increase in surface area means increasing the amount of exposed coordinately structural oxygen ions at the surface. However, the OSCs at 700°C are not greatly different from one another, which show the same tendency with H_2_ consumed. This suggests that the H_2_–TPR peak can be attributed to the reduction of lattice oxygen species, which may participate to the redox cycle at higher temperatures [27].

**Table 2:** H_2_–TPR and total OSC results.

	H_2_–TPR		OSC	
Samples	PeakTemperature(°C)	H_2_consumed	200°C	700°C
CO	603	20.6^a^ (1193.5)^b^	42.2^a^ (122.2)^b^	236^a^ (683.7)^b^
CO–SC	588	21.6^a^ (1251.4)^b^	108.9^a^ (315.5)^b^	245^a^ (709.7)^b^
ME	606	23.4^a^ (1355.7)^b^	75.3^a^ (218.1)^b^	273^a^ (790.8)^b^
ME–SC	586	24.3^a^ (1407.9)^b^	124.6^a^ (360.9)^b^	295^a^ (853.1)^b^

a µmol O_2_/g sample;

b µmol O_2_/g CeO_2_.

According to XPS compositional analysis (Table 3 and Figure S5), it is deduced that the SC process may bring about Ce enrichment, high adsorbed oxygen (O^−^, O_2_^2−^ and O_2_^−^) and Ce^3+^/Ce percentage on the surface, which is attributed to the high dispersion of nanoparticles. This should be another reason why the SC samples possess higher OSCs at lower temperatures in comparison with those using conventional drying method in air, because it is testified that only the Ce^3+^/Ce^4+^ plays the role of active redox couple in the Pr-doped CeZr (Once totally reduced, Pr^3+^ is difficult to be reoxidized [28]) and Nd-doped CeZr (Nd is a trivalent cation [29]) samples. As confirmed by XPS (Figure S5), both Pr and Nd are in the valence of +3.

**Table 3:** XPS analysis results.

	Surface atomic ratio (%)				O_II_ a (%)	Ce^3+^/Ce (%)
Sample	Ce	Zr	Pr	Nd		
CO	27.65	54.87	7.98	9.49	22.45	47.36
CO–SC	34.83	41.57	13.28	10.32	30.81	57.85
ME	27.85	52.86	10.95	8.33	24.44	52.55
ME–SC	31.05	47.79	12.63	8.45	30.34	57.09

a Adsorbed oxygen/(Adsorbed and lattice oxygen, Figure S5c).

In conclusion, the cubic Pr and Nd co-doped Ce–Zr oxide solid solutions were prepared by co-precipitation and microemulsion methods. The samples dried using supercritical CO_2_ show the significant improvement of specific surface areas and OSCs at lower temperatures after aging at 1000°C for 12 h in comparison with those using conventional air drying and even supercritical ethanol drying. Supercritical CO_2_ drying can result in the loosely aggregated morphology due to the elimination of surface tension effects on the gas-liquid interface and the resultant Ce enrichment on the nanoparticle surfaces.

## Supporting Information

Figure S1 **XRD pattern of CZ–0.75 (the molar ratio of Ce/Zr is 0.75) prepared by co-precipitation, conventional air drying and calcination at 1000°C for 12 h in air.**
(TIF)

Figure S2 **Le Bail fitting-patterns of (a) CO; (b) CO-SC; (c) ME; (d) ME-SC; (e) CZ-0.75.** Blue: experimental spectra; red: fitted spectra; gray: difference spectra.
(TIF)

Figure S3 **XRD (a) and N_2_ adsorption/desorption and pore size distribution curves (b) for CZPN oxide prepared by co-precipitation, supercritical ethanol drying and calcination at 1000°C for 12 h in air.** The XRD pattern (a) shows a cubic fluorite structure (JCPDS 38–1439).
(TIF)

Figure S4 **The first, second and third cycles of the H_2_-TPR profiles for CO, CO–SC, ME and ME–SC.**
(TIF)

Figure S5 **XPS spectra (a) of CO, CO–SC, ME and ME–SC; Ce 3d spectra (b):** Peaks denoted by v, v″, v″′, u, u″ and u″′ are characteristic peaks of Ce^4+^ ions, whereas those marked by v^0^, v, u^0^ and u, are of Ce^3+^ ions; **O 1 s spectra (c):** The signals at 529.5 eV (O_I_) and 531.5 eV (O_II_) are assigned to surface lattice oxygen and adsorbed oxygen species such as O^−^, O_2_^2−^ and O_2_^−^, respectively; **Zr 3 d spectra (d):** The Zr 3****d spectra belong to Zr^4+^; **Pr 3 d spectra (e):** m and m′: main peaks; s and s′: satellites; t′: extra structure existing only in 3 d 3/2component. The Pr 3 d spectra are quite similar to that of Pr_2_O_3_; **Nd 3** **d spectra (f):** The positions of Nd 3 d peaks indicate that the Nd is in the valence of +3.
(TIF)

Table S1 **Structural parameters as detected by the profile fitting of the XRD patterns of CO, CO-SC, ME, ME-SC, and CZ-0.75 using Le Bail method with the computer program TOPAS.**
(DOC)

Table S2 **Textural properties.**
(DOC)

Table S3 **Cyclic (1^st^/2^nd^/3^rd^) H_2_–TPR results.**
(DOC)
