# Supplementary figures and images for: Significant Improvement of Thermal Stability for CeZrPrNd Oxides Simply by Supercritical CO2 Drying
Source: PLoS One. 2014 Feb 7;9(2):e88236. doi: 10.1371/journal.pone.0088236 (PMC3917872; doi:10.1371/journal.pone.0088236)

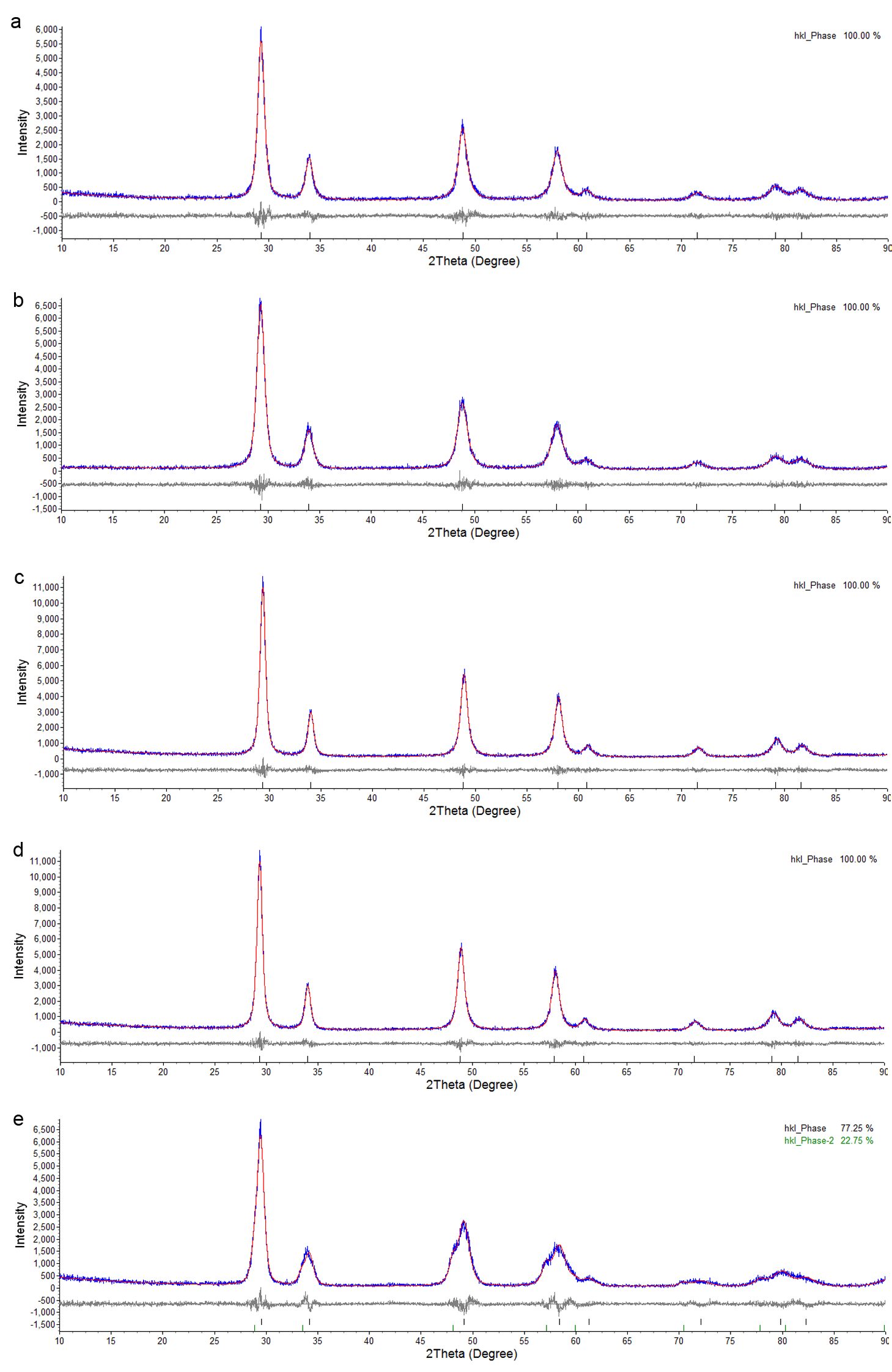

Supplement: Figure S2 — Le Bail fitting-patterns of (a) CO; (b) CO-SC; (c) ME; (d) ME-SC; (e) CZ-0.75. Blue: experimental spectra; red: fitted spectra; gray: difference spectra. (TIF) [file pone.0088236.s002.tif]

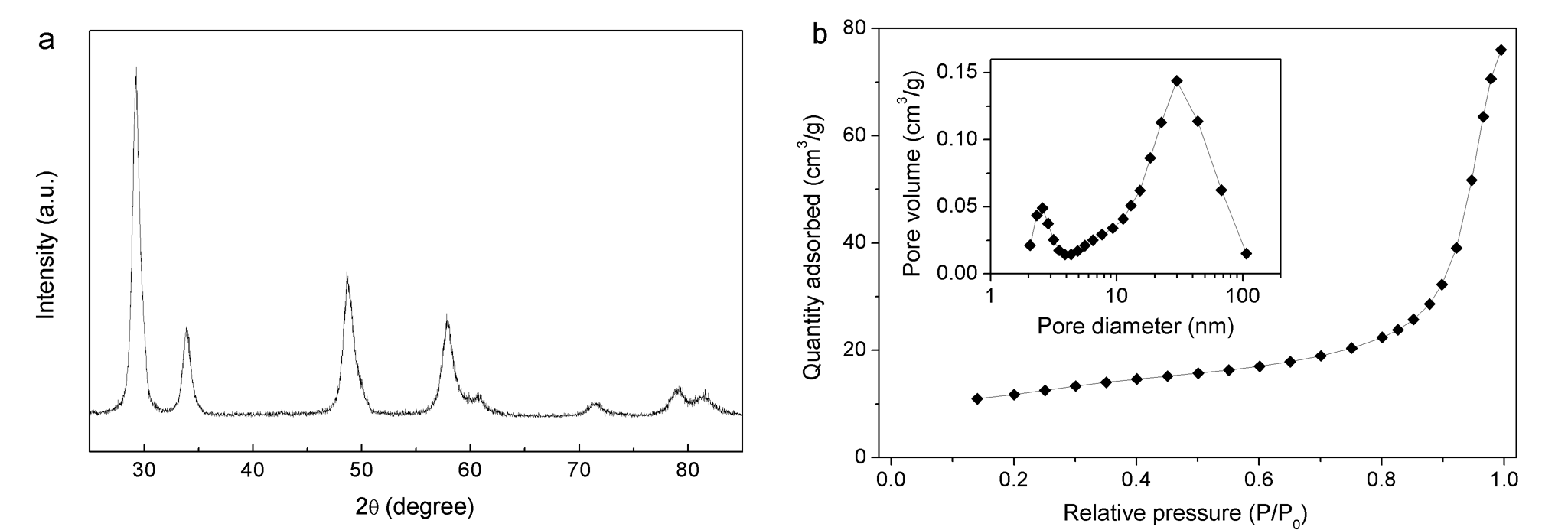

Supplement: Figure S3 — XRD (a) and N2 adsorption/desorption and pore size distribution curves (b) for CZPN oxide prepared by co-precipitation, supercritical ethanol drying and calcination at 1000°C for 12 h in air. The XRD pattern (a) shows a cubic fluorite structure (JCPDS 38–1439). (TIF) [file pone.0088236.s003.tif]

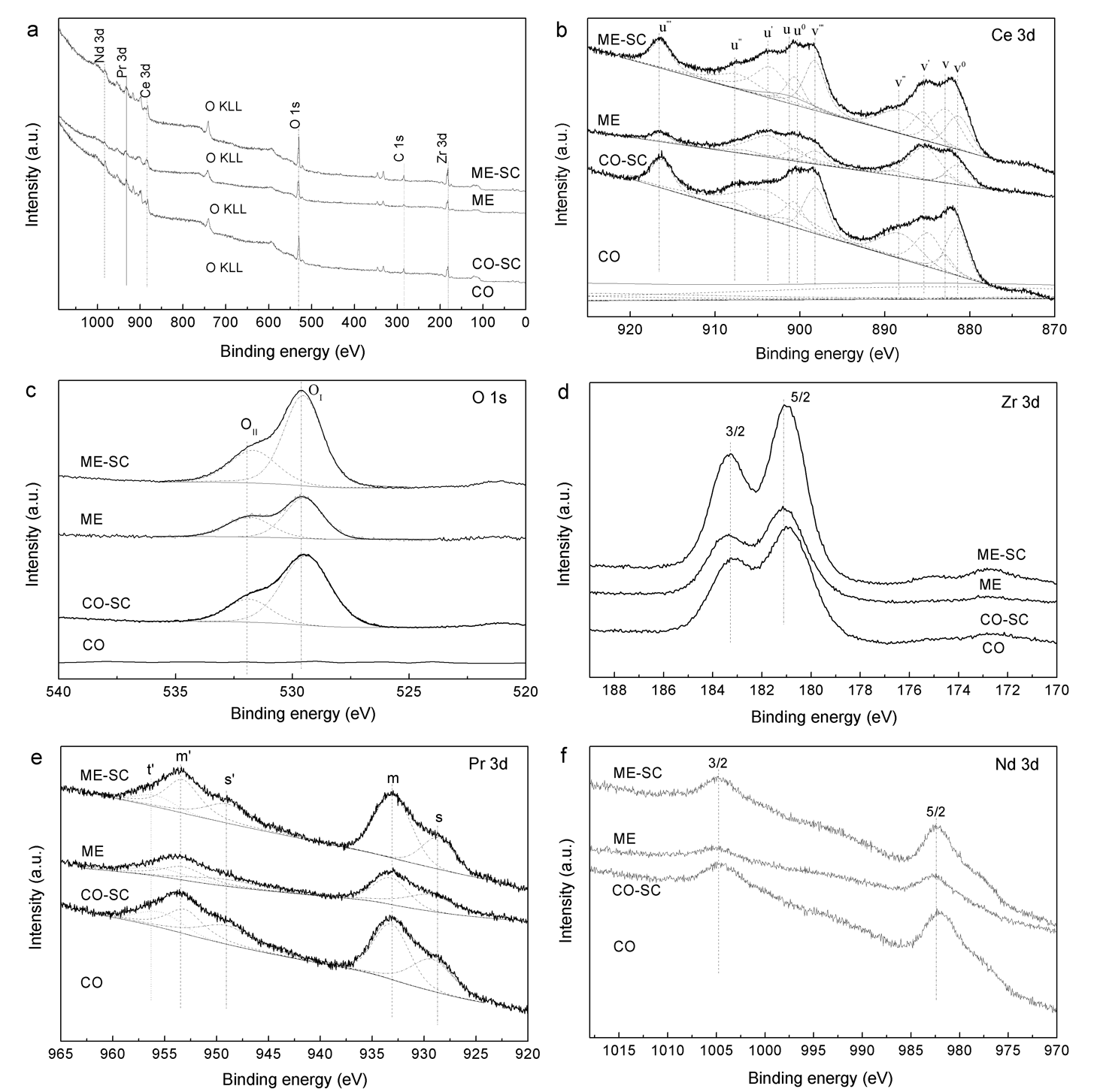

Supplement: Figure S5 — XPS spectra (a) of CO, CO–SC, ME and ME–SC; Ce 3d spectra (b): Peaks denoted by v, v″, v″′, u, u″ and u″′ are characteristic peaks of Ce4+ ions, whereas those marked by v0, v, u0 and u, are of Ce3+ ions; O 1 s spectra (c): The signals at 529.5 eV (OI) and 531.5 eV (OII) are assigned to surface lattice oxygen and adsorbed oxygen species such as O−, O2 2− and O2 −, respectively; Zr 3 d spectra (d): The Zr 3d spectra belong to Zr4+; Pr 3 d spectra (e): m and m′: main peaks; s and s′: satellites; t′: extra structure existing only in 3 d 3/2component. The Pr 3 d spectra are quite similar to that of Pr2O3; Nd 3 d spectra (f): The positions of Nd 3 d peaks indicate that the Nd is in the valence of +3. (TIF) [file pone.0088236.s005.tif]
