# Supplementary material for: Significant Improvement of Thermal Stability for CeZrPrNd Oxides Simply by Supercritical CO2 Drying
Source: PLoS One. 2014 Feb 7;9(2):e88236. doi: 10.1371/journal.pone.0088236 (PMC3917872; doi:10.1371/journal.pone.0088236)
Supplement: Table S1 — Structural parameters as detected by the profile fitting of the XRD patterns of CO, CO-SC, ME, ME-SC, and CZ-0.75 using Le Bail method with the computer program TOPAS. (DOC) [file pone.0088236.s006.doc]

**Table S1. Structural parameters as detected by the profile fitting of the XRD patterns of CO, CO-SC, ME, ME-SC, and CZ-0.75 using Le Bail method with the computer program TOPAS.**

| **Samples** | **Crystalline phase** | **Space group** | **Phase content (wt%)** | **R_p_ (%)** | **R_wp_ (%)** | **R_exp_ (%)** | **GOF** |
| --- | --- | --- | --- | --- | --- | --- | --- |
| CO | Ce_0.6_Zr_0.4_O_2_ | Fm-3m | 100 | 12.18 | 17.71 | 5.73 | 3.09 |
| CO–SC | Ce_0.6_Zr_0.4_O_2_ | Fm-3m | 100 | 11.20 | 17.04 | 5.47 | 3.11 |
| ME | Ce_0.6_Zr_0.4_O_2_ | Fm-3m | 100 | 9.02 | 13.19 | 4.25 | 3.10 |
| ME–SC | Ce_0.6_Zr_0.4_O_2_ | Fm-3m | 100 | 9.04 | 13.25 | 4.25 | 3.12 |
| CZ-0.75^a^ | Ce_0.6_Zr_0.4_O_2_ | Phase I: Fm-3m | 77.25 | 11.79 | 16.86 | 4.99 | 3.38 |
|  | Ce_0.75_Zr_0.25_O_2_ | Phase II: Fm-3m | 22.75 |  |  |  |  |

a: Phase I: Ce_0.6_Zr_0.4_O_2_ (JCPDS 38-1439); phase II: Ce_0.75_Zr_0.25_O_2_ (JCPDS 28-0271).
