# Supplementary material for: Significant Improvement of Thermal Stability for CeZrPrNd Oxides Simply by Supercritical CO2 Drying
Source: PLoS One. 2014 Feb 7;9(2):e88236. doi: 10.1371/journal.pone.0088236 (PMC3917872; doi:10.1371/journal.pone.0088236)
Supplement: Table S2 — Textural properties. (DOC) [file pone.0088236.s007.doc]

**Table S2. Textural properties.**

| **Samples** | **BET surface area (m^2^/g)** | **Pore volume (cm^3^/g)** | **Average pore diameter (nm)** |
| --- | --- | --- | --- |
| CZ–0.75 | 0.02 | 0.0006 | 43.9 |
| CZPN oxide prepared by co-precipitation followed by supercritical ethanol drying | 40.4 (27.6)^a^ | 0.115 (0.006)^b^ | 13.8 |

a: t-plot external surface area; b: t-plot micropore volume. Supercritical ethanol drying conditions: 250 °C, 6.4 MPa.
