# Supplementary material for: Significant Improvement of Thermal Stability for CeZrPrNd Oxides Simply by Supercritical CO2 Drying
Source: PLoS One. 2014 Feb 7;9(2):e88236. doi: 10.1371/journal.pone.0088236 (PMC3917872; doi:10.1371/journal.pone.0088236)
Supplement: Table S3 — Cyclic (1st/2nd/3rd) H2–TPR results. (DOC) [file pone.0088236.s008.doc]

**Table S3. Cyclic (1^st^/2^nd^/3^rd^) H_2_–TPR results.**

| **Samples** | **Peak temperature (°C)** | **H_2_ consumed** |
| --- | --- | --- |
| CO | 606/604/606 | 20.6^a^ (1193.5)b/20.5 (1187.7)/20.2 (1170.3) |
| CO–SC | 590/588/588 | 21.4^a^ (1239.8)b/21.3 (1234.1)/21.2 (1228.2) |
| ME | 609/609/607 | 23.2^a^ (1344.1)b/23.2 (1344.1)/23.1 (1338.3) |
| ME–SC | 587/587/586 | 24.2^a^ (1402.1)b/24.2 (1402.1)/24.0 (1390.5) |

a: μmol O_2_/g sample; b: μmol O_2_/g CeO_2_.
